# Neoadjuvant vs. Adjuvant Chemotherapy in Muscle Invasive Bladder Cancer (MIBC): Analysis From the RISC Database

**DOI:** 10.3389/fonc.2018.00463

**Published:** 2018-11-19

**Authors:** Gabriella Del Bene, Fabio Calabrò, Diana Giannarelli, Elizabeth R. Plimack, Lauren C. Harshman, Evan Y. Yu, Simon J. Crabb, Sumanta Kumar Pal, Ajjai S. Alva, Thomas Powles, Ugo De Giorgi, Neeraj Agarwal, Aristotelis Bamias, Sylvain Ladoire, Andrea Necchi, Ulka N. Vaishampayan, Günter Niegisch, Joaquim Bellmunt, Jack Baniel, Matthew D. Galsky, Cora N. Sternberg

**Affiliations:** ^1^San Camillo-Forlanini Hospital, Rome, Italy; ^2^IRCCS-Regina Elena Cancer Institute, Rome, Italy; ^3^Fox Chase Cancer Center, Philadelphia, PA, United States; ^4^Dana-Farber Cancer Institute, Harvard Medical School, Boston, MA, United States; ^5^Fred Hutchinson Cancer Research Center, University of Washington, Seattle, WA, United States; ^6^University of Southampton, Southampton, United Kingdom; ^7^City of Hope Comprehensive Cancer Center, Duarte, CA, United States; ^8^University of Michigan, Ann Arbor, MI, United States; ^9^St. Bartholomew's Hospital, London, United Kingdom; ^10^Istituto Scientifico Romagnolo per lo Studio e la Cura dei Tumori, IRCCS, Meldola, Italy; ^11^Huntsman Cancer Institute at the University of Utah, Salt Lake, UT, United States; ^12^National & Kapodistrian University of Athens, Athens, Greece; ^13^Georges François Leclerc Cancer Center, Dijon1, France; ^14^Istituto Nazionale Tumori of Milan, Milan, Italy; ^15^Karmanos Cancer Institute, Detroit, MI, United States; ^16^Department of Urology, Medical Faculty, Heinrich-Heine-University, Düsseldorf, Germany; ^17^Rabin Medical Center, Petah Tikva, Israel; ^18^The Tisch Cancer Institute, Icahn School of Medicine at Mount Sinai, New York, NY, United States

**Keywords:** muscle invasive bladder cancer, neoadjuvant chemotherapy, adjuvant chemotherapy, RISC data base, locally advanced bladder cancer

## Abstract

**Background:** MIBC is an aggressive disease, with 5-year survival rates ranging from 36 to 48% for p T3/p T4/p N+tumors. Perioperative treatment can improve overall survival, with more robust evidence in favor of neoadjuvant chemotherapy. Few randomized studies have compared neoadjuvant and adjuvant therapy in bladder cancer. Consequently, it has been difficult to establish the benefit of adjuvant chemotherapy (AC) in MIBC.

**Methods:** Data from patients with muscle invasive bladder cancer (>pT2) collected from 2005 to 2012 within the RISC data base (Retrospective International Study of Cancers of the Urothelial Tract) were evaluated. Overall survival (OS), cancer specific survival (CSS), and disease-free survival (DFS) between NC and AC generated using the Kaplan-Meier method were compared for MIBC by log-rank test. All patients in this analysis received either NC or AC.

**Results:** A total of 656 patients with MIBC (325 treated with AC and 331 with NC) were analyzed. The median DFS was 34.6 months (95% CI:25.3–43.9) for NC vs. 24.9 months (95% CI: 19.4–30.5) with AC, with a reduction in the risk of disease progression of 21% in favor of NC (HR: 0.78, 95% CI: 0.63–0.96, *P* = 0.02). There were no significant differences in terms of CSS (HR: 1.06, 95% CI: 0.79–1.43, *P*: 0.70), and OS (HR: 1.08, 95% CI: 0.83–1.39, *P* = 0.57).

**Conclusions:** This study demonstrates superiority in DFS for NC compared to AC. The positive prognostic impact of complete pathological response to NC was confirmed.

## Introduction

It is estimated that about 1.7 million new cases of cancer will be diagnosed in 2018 in the US with bladder cancer being the fourth most common cancer within males (1).

At the time of diagnosis, about 75–80% of bladder cancers are superficial, while the remaining 15–20% present as muscle invasive tumors. Approximately 50% of patients develop metastatic disease and in the past the median overall survival was dismal at 3–6 months without systemic treatment. The addition of cisplatin based therapy improved survival to between 12 and 15 months (2).

Despite improvement in surgical techniques, the rate of local and remote relapse remains high. The 5-year overall survival rates after radical cystectomy range from 36 to 48% for pT3-T4 and/or pN0/pN+ disease, most likely due to the presence of micro metastasis at the time of diagnosis (3).

Perioperative treatments either before or after surgery, can reduce the risk of both local and distant recurrence and increase overall survival.

Several studies and meta-analyses have been conducted, with more consistent results in favor of NC therapy, which is recommended for the treatment of MIBC (level 1 evidence). The lack of robust evidence for AC derives mainly from the difficulties in accrual and methodological problems of the trials that have been conducted in this setting. However, AC represents an important option for patients with MIBC (pT3-T4 and/orpN0 /pN+ disease) who have not received NC.

Few studies, mostly retrospective, have compared the two treatment strategies of NC and AC, so data on the optimal sequence remains controversial. The aim of this analysis is to compare the efficacy of NC and AC treatment in MIBC, based on data from the RISC database.

## Materials and methods

The Retrospective International Study of Cancers of the Urothelial Tract (RISC) is a population-based, retrospective study with the primary objective to describe the management, patterns of care and outcome of patients with urothelial cancer (clinical T-classification cT2 disease or greater). RISC consists of patient series from 28 international centers between 2005 and 2012 (some centers included patient data over this entire period, whereas others included data over a more limited number of years). Data concerning baseline characteristics, laboratory and pathology information, and treatment outcomes were collected using a password-protected, secure, web-based, electronic data capture tool. Investigators were trained using a web-based tutorial and supported by a comprehensive study training manual and data dictionary. The coordinating center, Icahn School of Medicine, Mount Sinai Hospital, New York, NY, curates the data with queries completed by each participating site. The study was approved by the ethics committees at each participating institution.

Patient inclusion criteria for the current analysis included a diagnosis of muscle invasive bladder cancer of any histology treated with NC (≥cT2, cN0, M0) followed by surgery (radical cystectomy), or the contrary, with cystectomy first, followed by AC (≥pT2, any pN, M0). Patients treated with adjuvant radiotherapy or a combination of radiation and chemotherapy were excluded, as well as those treated with both NC and AC.

The primary objectives were the comparison between NC and AC, in terms of disease free survival (DFS), overall survival (OS), and cancer specific survival (CSS), which were calculated from the time of diagnosis of muscle invasive disease until disease progression, death from any cause or specifically from cancer, respectively. DFS, OS, and CSS were calculated with 95% confidence intervals, with analysis of survival generated using the Kaplan-Meier method. The comparison between survival curves for every endpoint, was done with the log rank test (4). Hazard ratios and their 95% CI were derived from the proportional hazard model and were estimated for type of chemotherapy (NC vs. AC), histology (urothelial vs. non-urothelial), pT and pN status, Charlson index, age, gender, and smoking habits. A propensity matched analysis was also performed, matching samples according to pT and pN status.

There is an issue about what to consider as time 0 for this kind of analysis. If defined by the initiation of chemotherapy, the NC patients would necessarily have had to live longer than AC patients. That is, NC patients by definition had to live about 3–4 months longer because their time 0 was the start of NC, and by definition they had to survive to have cystectomy. On the other hand, time 0 for the AC patients would start months later at the start of chemotherapy.

At the same time, if it is considered the date of cystectomy as time 0, it still may lead to an unfair advantage for the adjuvant patients: because the receipt of AC occurs after cystectomy and patients in the AC group are defined by the receipt of AC. This group by definition still has to live long enough to receive AC and longer than NC group. So, this can deprive the NC group of 3–4 months of survival and does not allow those who did not undergo radical cystectomy to be considered in calculations of survival rates. For this reason, we felt that the soundest methodology for this type of analysis was to use time of diagnosis of bladder cancer as time 0 for both NC and AC.

## Results

From 2005 to 2012 a total of 3,024 patients with urothelial cancer were included in the RISC database. Overall, 1,892 of these patients underwent surgery for MIBC and UTUC. Seventy-four patients were excluded from the original sample due to lack of data, 139 due to incomplete data concerning perioperative therapy, 39 patients with lymph node metastatic disease (cN+) were excluded from the NC group, 875 were not considered because they had not received any perioperative treatment, while 32 were left out of the analysis because they underwent both treatments (NC and AC).

**Figure 1:**
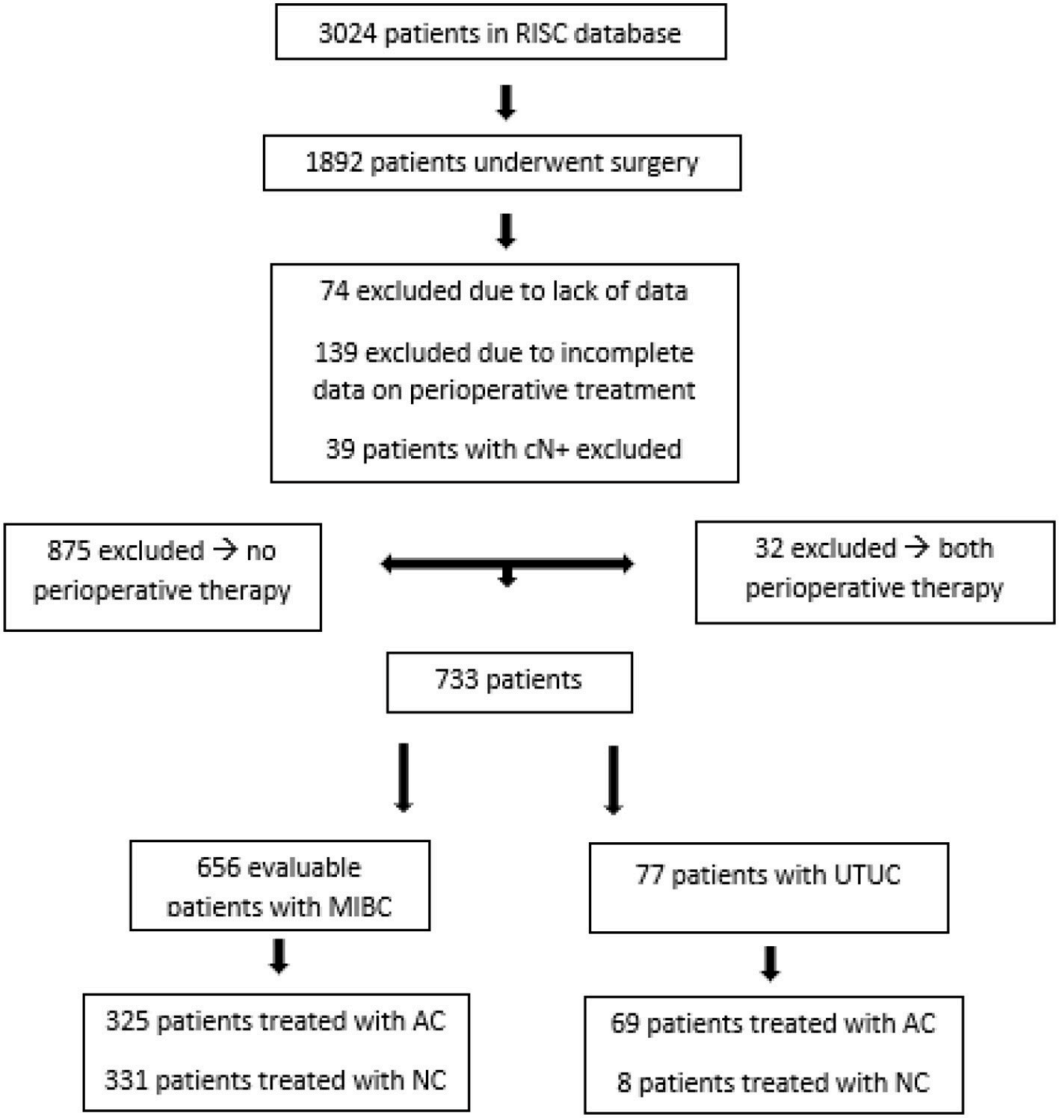
Flow chart: patients captured from the RISC database. UTUC, Upper tract urothelial carcinoma. This group was not included in this analysis.

Therefore, the final sample for the analysis consists of 656 patients with MIBC, treated with surgery and perioperative chemotherapy. Among patients with MIBC, 325 were treated with AC and 331 with NC (Figure 1).

Patient characteristics are detailed in Table 1. The majority of patients (>70%) were male. Other patient characteristics, in particular with regard to age (< 65 years or ≥65 years) and Charlson index, were equally distributed among the various groups.

**Table 1:** Patient characteristics.

**N^°^ of patients (%)**	**NC MIBC (331)**	**AC MIBC (325)**	***P*-value**
**GENDER**			
Male	257 (77.6)	271 (83.4)	0.06
Female	74 (22.4)	54 (16.6)	
**AGE**			
< 65 years	179 (54.1)	184 (56.6)	0.23
≥65 years	145 (43.8)	139 (42.8)	
missing	7 (2.1)	2 (0.6)	
**CHARLSON**			
0	154 (46.5)	133 (41.0)	0.34
1–2	90 (27.2)	107 (32.9)	
≥3	61 (18.4)	56 (17.2)	
Missing	26 (7.9)	29 (8.9)	
**SMOKING HISTORY**			
Current	76 (30.0)	83 (25.5)	0.29
Former	140 (42.3)	131 (40.3)	
Never	89 (26.9)	72 (22.2)	
Missing	29 (8.8)	39 (12.0)	
**pT**			
0	91 (27.5)	3 (0.9)	< !0.0001
1	16 (4.8)	6 (1.8)	
2	56 (16.9)	55 (16.9)	
3	87 (26.3)	165 (50.8)	
4	35 (10.6)	77 (23.7)	
Ta	6 (1.8)	1 (0.3)	
Tis	21 (6.3)	1 (0.3)	
Missing	19 (5.7)	17 (5.3)	
**pN**			
0	234 (70.7)	74 (22.8)	< !0.0001
+	68 (20.5)	214 (65.8)	
x	9 (2.7)	14 (4.3)	
Missing	20 (6.1)	23 (7.1)	
**cT**			
0	0	1 (0.3)	0.01
1	6 (1.8)	15 (4.6)	
2	183 (55.3)	169 (52.0)	
3	83 (25.1)	75 (23.1)	
4	29 (8.8)	18 (5.6)	
Ta	0	2 (0.6)	
Tis	0	1 (0.3)	
Missing	30 (9.1)	44 (13.5)	
**cN**			
0	219 (66.1)	135 (41.6)	< !0.0001
+	0	69 (21.2)	
x	79 (23.9)	69 (21.2)	
Missing	33 (10.0)	52 (16.0)	
**HISTOLOGY**			
TCC	237 (71.6)	248 (76.3)	0.10
TCC+other	44(13.3)	28 (8.6)	
Other	35 (10.6)	41 (12.6)	
Missing	15 (4.5)	8 (2.5)	
**CT REGIMEN**			
Cisplatin based	248 (74.9)	231 (71.1)	0.18
No CDDP based	73 (22.1)	75 (23.1)	
Missing	10 (3.0)	19 (5.8)	

*TCC, Transitional cell carcinoma; CT, chemotherapy; CDDP, cisplatin. Other (non-urothelial histologies: adenocarcinoma, squamous cell carcinoma, sarcomatoid carcinoma, small cell carcinoma, pure micropapillary, urachal adenocarcinoma)*.

The significant difference relative to the percentage of patients with clinically positive lymph nodes among the two groups, derives mainly from the need to exclude those patients from the NC group, as they had clear metastatic disease at the time of diagnosis and they should have been treated with more cycles of chemotherapy. Moreover, the differences between the pT and pN in the two groups may be due to down-staging as a result of neoadjuvant chemotherapy.

More than 70% of patients were smokers or former smokers, and most of them had pure urothelial histology (>70%) in each group. Mixed histology (urothelial with other features: squamous, neuroendocrine, sarcomatoid, micropapillary, etc.) was observed in 21.9% of patients. Non-urothelial histologies were found in 23.2%of patients.

The majority of patients, both in the NC and AC setting, were treated with combination cisplatin based chemotherapy. In particular, in the MIBC NC group, 74.9% of patients were treated with cisplatin based chemotherapy, while 22.1% received treatment without cisplatin. In the MIBC AC group, 71.1% received cisplatin based therapy vs. 23.1% non-cisplatin based. The different chemotherapy regimens used are shown in the Appendix in Supplementary Material.

**Figure 2:**
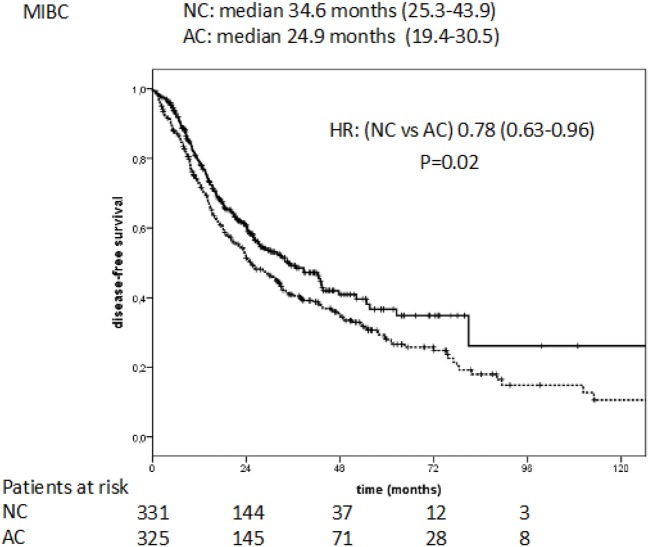
Disease free survival in NC vs. AC treated patients with MIBC.

The median DFS was 34.6 months (95% CI: 25.3–43.9) for NC vs. 24.9 months (95% CI: 19.4–30.5) with AC, with a reduction in the risk of disease progression of 21% in favor of NC (HR: 0.78, 95% CI: 0.63–0.96, *P* = 0.02; Figure 2).

**Figure 3:**
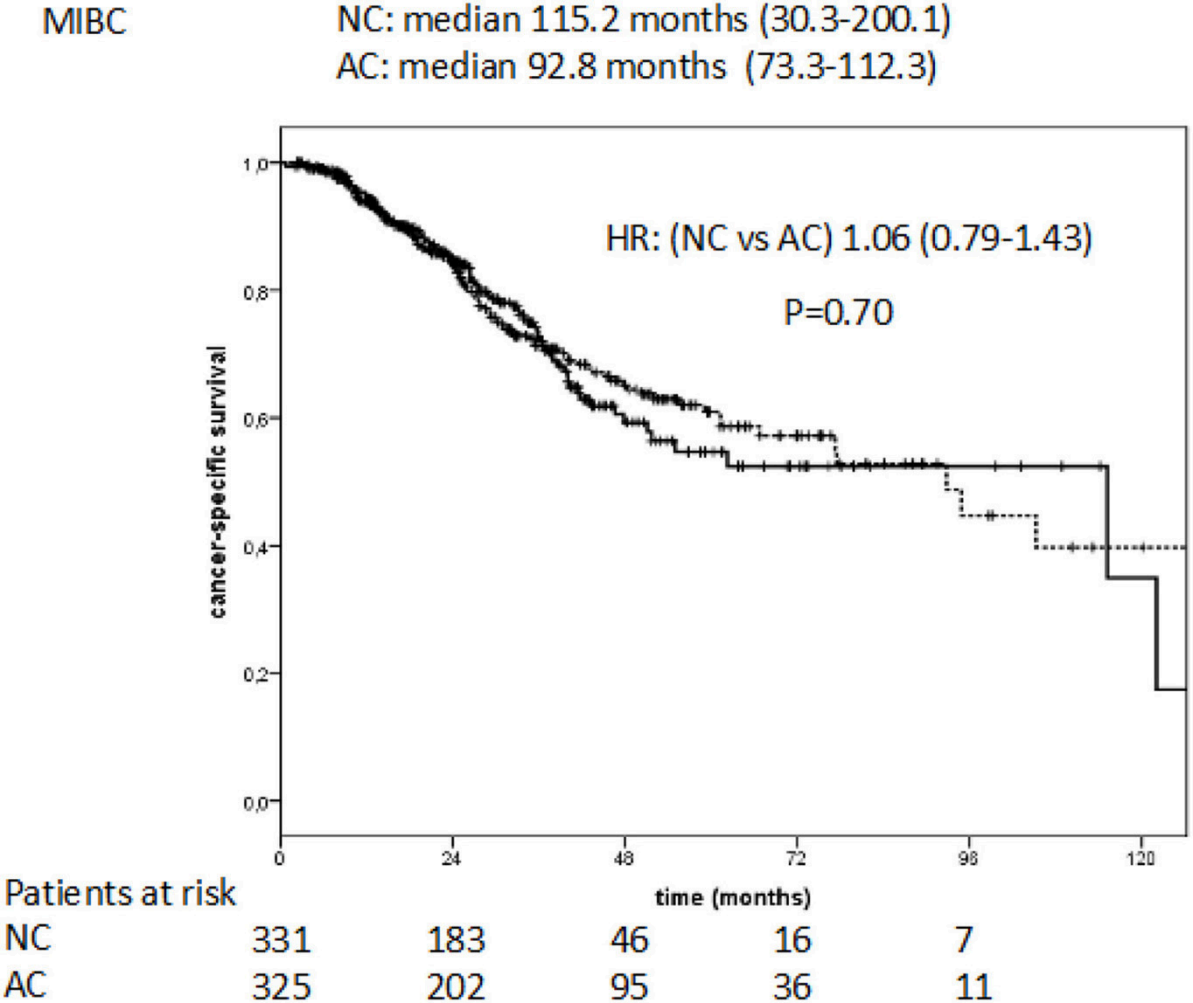
Cancer specific survival in NC vs. AC patients with MIBC.

The median cancer specific survival (CSS) was 115.2 months (95% CI: 30.3–200.1) for NC vs. 92.8 months (95% CI: 73.3–112.3) for AC. This difference was not statistically significant (HR: 1.06, 95% CI: 0.79–1.43, *P* = 0.70; Figure 3).

**Figure 4:**
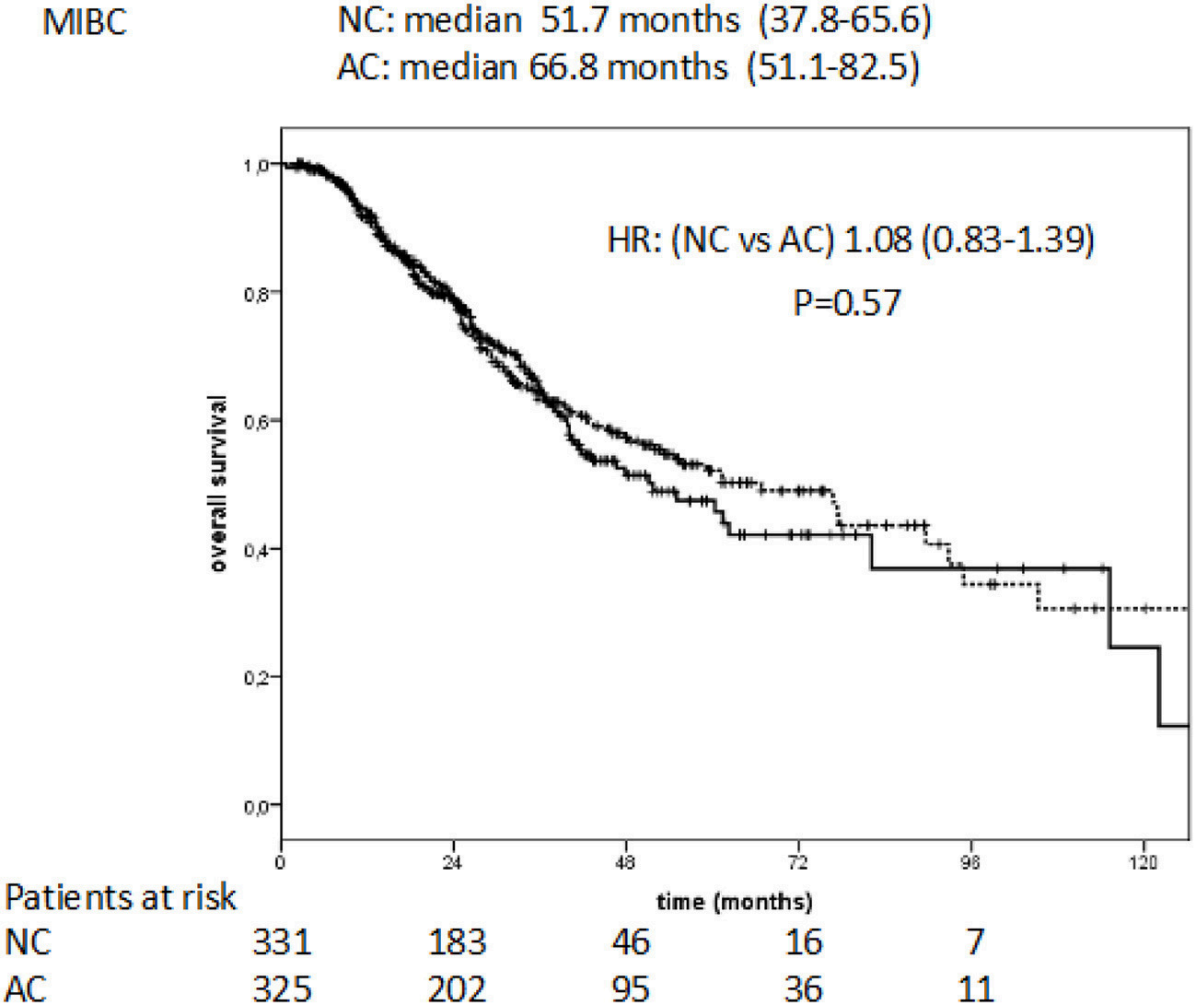
Overall survival in NC vs. AC treated patients with MIBC.

The median overall survival (OS) was of 51.7 months (95% CI:37.8–65.6) for the NC group and 66.8 months (95% CI: 51.1–82.5) for AC. The difference was not statistically significant (HR: 1.08; 95 % CI 0.83–1.39, *P* = 0.57; Figure 4).

There was a significant difference in DFS and OS between patients with complete pathologic response (pT0) after NC compared to those with residual disease (≥pT1; DFS at 5 years pT0 vs. ≥pT1: 62 vs. 21%, respectively, *P* < 0.0001; OS at 5 years pT0 vs. ≥pT1: 72 vs. 31%, respectively, *P* < 0.0001; Appendix, Supplementary Survival Curves).

There was longer DFS and OS for NC in MIBC for patients without lymph node metastasis, compared to those with lymph node involvement (DFS 5 years pN0 vs. pN+: 47 vs. 9%, respectively, *P* < 0.0001; OS 5 years pN0 vs. pN+: 51 vs. 25%, respectively, *P* < 0.0001; Appendix, Supplementary Survival Curves).

In the AC group, there was a significant difference in DFS (*P* = 0.001) and OS (*P* < 0.0001) at 5 years in relation to pT status, with better results for lower stages than more advanced stages (pT2 vs. pT3 vs. pT4), while there was no significant difference for DFS (*P* = 0.11) or OS (*P* = 0.29) in relation to pathological lymph node status (pN0 vs. pN+).

In the AC group, there was also better OS at 5 years for urothelial histology than for non-urothelial tumors, (56 vs. 43%, *P* = 0.001), significant differences were observed also in DFS at 5 years (31 vs. 19%, *P* = 0.002).

A multivariate analysis of survival times (DFS, CSS, OS) was performed. The variables considered in the Cox regression model were sex, age, T, N, smoking habits, Charlson index, histology, and NC vs. AC. The hazard ratio of NC vs. AC revealed a positive effect on DFS for NC with respect to AC only in the univariate analysis, not in the multivariate analysis, and a negative effect for CSS and OS. A complete pathological response and the absence of lymph node metastasis had a significant positive impact on DFS, OS, and CSS in both multivariate and univariate analysis (Supplementary Table 1). In the multivariate analysis, the HRs (adjusted for pN and pT) were in favor of AC for all the three survival endpoints.

When considering a propensity score based on the two prognostic factors (pT and pN) differences between NC and AC in DFS disappeared (*P* = 0.38); also differences in terms of CSS (*P* = 0.40) and OS (*P* = 0.26) resulted not significant.

## Discussion

Muscle invasive bladder cancer is a highly aggressive disease, with a high rate of early metastatic dissemination, and a low 5-year overall survival rate. Perioperative cisplatin-based chemotherapy in addition to surgery to improve outcomes in high risk MIBC has shown increased disease control with improved survival, probably due to eradication of micrometastatic disease. Currently, the evidence favors NC, which has shown an advantage in terms of OS for cisplatin based combinations in at least two randomized trials and multiple meta-analyses.

In particular, the MRC BA06/EORTC 30894 trial, demonstrated a 10-year benefit of 6% for NC, and the SWOG study by Grossmann et al, confirmed a trend toward better OS in favor of NC with M-VAC (5, 6). Moreover, in 2005, a meta-analysis of 11 trials of NC encompassing 3,005 patients showed a reduction in the risk of death of 14% (HR = 0.86, 95% CI 0.77–0.95, *P*: 0.003), with a survival advantage at 5 years of 5% (from 45 to 50%) (7).

The role of AC is more controversial given data derived mainly from studies with inconsistent results due to methodological problems and inadequate patient numbers due to premature closure and poor recruitment. No single study, taken individually, has demonstrated a statistically significant benefit in survival in favor of AC with the exception of the Spanish study (Spanish Oncology Genitourinary Group-SOGUG). It showed a potential benefit in OS for AC with cisplatin, paclitaxel, gemcitabine (median OS 26 months; 5-year OS, 31%; *P* < 0.0009), DFS (*P*: < 0.0001), TTP (*P*: < 0.0001), and CSS (*P*: < 0.0002). This study was closed prematurely due to poor recruitment, and the results were presented at an ASCO meeting but have never been fully published (8).

The EORTC performed the largest AC study to date (trial 30994). This study was also hampered by difficulties in enrolment. The study evaluated immediate AC with four cycles of chemotherapy vs. observation and six cycles of chemotherapy at the time of recurrence. GC, M-VAC or HD-VAC were allowed. This study did not show a benefit in OS (adjusted HR 0.78, 95% CI.0.56–1.08; *P* = 0.13), but did reveal a highly significant improvement in PFS, with 5 year PFS of 47.6 vs. 31.8% for those given immediate adjuvant chemotherapy (HR: 0.54, 95% CI: 0.40–0.73, *P* < 0.0001). There was, however, a benefit in OS for the subgroup of patients with negative lymph nodes (pN0) (9) and a non-significant 22.2% reduction in the risk of death with immediate adjuvant chemotherapy in the ITT population.

Meta-analyses have been conducted to clarify the role of AC. The analysis published in 2014 without the EORTC study data, showed a reduction in the risk of death with AC of 23% (HR 0.77, 95% CI 0.59–0.99; *P*: 0.049) (10). A further update of this meta-analysis, conducted with the EORTC 30994 study, demonstrated a survival benefit with immediate adjuvant treatment (HR 0.77, 95% CI 0.65–0.91; *P* = 0.002) (10, 11).

Therefore, at present cisplatin-based combination AC is a valuable option for patients with bladder cancer pT3-pT4, pN0/pN+, M0 who have not received preoperative treatment. Currently, it is not possible to establish with absolute certainty what is the best sequence of perioperative treatments. The most persuasive evidence has been in favor of NC.

Until now, only one study compared NC with AC in a prospective manner. This study was conducted in 2001 at the MD Anderson Cancer Center. In this trial, 140 patients were randomized to receive preoperative treatment with two cycles of M-VAC followed by surgery and three additional M-VAC cycles, or immediate surgery followed by five AC cycles. At a median follow-up of 6.8 years, no statistically significant differences were observed in OS and DSS between the two treatment groups (12).

In a retrospective study at Columbia University, OS and DSS were analyzed in 146 patients who received perioperative therapy between 1988 and 2009 (73 neoadjuvant and 73 adjuvant) (11).

In this report, no statistically significant difference between the two treatments was observed. Another retrospective study in 42 patients, compared the combination of cisplatin and gemcitabine in the NC and AC setting without demonstrating any difference in recurrence free survival (*P*: 0.124) (13). All of these results seem to suggest that the sequence of treatments surrounding cystectomy is less critical than the perioperative therapy itself.

The results of a retrospective study from the National Cancer Database were presented at the ASCO meeting in 2016. This study, based on a series of more than 1,600 patients treated with NC and 800 with AC, compared NC to AC and to surgery alone, in terms of OS. Multivariate analysis showed higher OS (*P*: 0.008) for the patients treated with NC (14). These results are not conclusive given the retrospective nature of the work, but may suggest further caution in interpretation of the results of meta-analysis and large retrospective studies in favor of the role of AC (10, 15).

In our retrospective study, the comparison between NC and AC was done in a broader sample of patients compared to studies conducted so far, second only to the National Cancer Data base study in size, and with a similar distribution of patients between the two treatments. Of 656 patients with MIBC of various histologies, 325 were treated with AC and 331 with NC.

Our analysis shows a statistically significant difference in DFS in favor of NC (HR: 0.78, 95% CI: 0.63–0.96, *P* = 0.02), without any significant advantage in CSS (HR: 1.06, 95% CI: 0.79–1.43, *P* = 0.70) and OS (HR: 1.08; 95 % CI 0.83–1.39, *P* = 0.57).

A possible explanation for the better DFS in the NC group may derive from the different pathological characteristics of the two groups. Indeed, in the adjuvant group, a significant percentage of patients had positive lymph nodes at diagnosis (cN+: 21.2%) and 21.2% of patients underwent surgery without data about lymph node status (cNx: 21.2%). This could justify the worse DFS of the AC group, probably due both to inadequate staging and to greater disease burden, as these patients have metastatic disease at the time of surgery and diagnosis.

The advantage in DFS does not translate into a benefit in CSS and OS. The absence of a significant difference in OS and CSS may derive from the not-negligible percentage of patients with positive lymph nodes in the NC group (pN+: 20.5%) and the number of patients without adequate pathological lymph node staging (pNx: 2.7%). These patients may have a poorer outcome due to the lack of chemotherapy responsiveness, micrometastatic disease that would later progress or a greater disease burden. Indeed, many of these patients with pN+ and pNx disease may have been under staged, and therefore not adequately treated.

Furthermore, the significance of DFS may be due to the higher number of events with respect to the number of deaths from disease considered in the CSS. When performing the Propensity Score matched for pT and pN all differences disappeared and NC and AC had a similar outcome.

Moreover, the presence of lymph node metastasis had a negative impact on outcomes in the NC group, with worse 5 year DFS and OS (DFS 5 years pN0 vs. pN+: 47 vs. 9%, respectively, *P* < 0.0001; OS 5 years pN0 vs. pN+: 51 vs. 25%, respectively, *P* < 0.0001). In the AC group, there are no significant differences in DFS (*P* = 0.11) or OS (*P* = 0.29) in relation to pathological lymph node status (pN0 vs. pN+).These results are not aligned with those of the EORTC adjuvant study, where the absence of lymph node involvement (p N0) was associated with better OS compared to patients with positive lymph nodes (pN+).

Similar to past studies, we observed that pathological complete response to NC (ypT0) positively influenced both DFS and OS at 5 years compared to patients with residual disease (≥pT1; *P*: < 0.0001). The percentage of complete pathological response (pCR = ypT0) in the NC group was 27.5% slightly less than that in the pivotal SWOG study (pCR: 38%), but aligned with other cisplatin-based NC prospective and retrospective studies which have ranged from ~20 to 38% (15–17).

Whether to give NC or AC to variant histologies is a clinically relevant question. In our study, variant histology and mixed forms (urothelial with a squamous component or other types) did not appear to affect 5-year DFS and 5-year OS in the NC (*P*: 0.15). However, in the AC group, patients with urothelial cancers did appear to have improved 5-year OS (56 vs. 43%, *P* = 0.001) and 5-year DFS (31 vs. 19%, *P* = 0.002) compared to patients with non-urothelial tumors.

In a secondary analysis of the SWOG S8710 study, the efficacy of NC was estimated in relation to histology, divided into urothelial tumors and mixed forms. There was a survival benefit for NC in patients with mixed histology (HR: 0.46, 95% CI 0.25–0.87, *P*: 0.02), as well as in those with urothelial tumors, although not statistically significant (HR: 0.90; 95% CI 0.67–1.21; *P*: 0.48) (18). These results, together with those of our study, suggest that the presence of a non-urothelial component may not confer resistance to chemotherapy, and does not constitute an element of absolute exclusion from perioperative treatment. Better 5-year DFS and OS were /not was not obtained with urothelial tumors in the AC group. Given the retrospective nature of the analysis, this cannot lead to definitive conclusions.

Our study is one of the largest global analyses conducted so far comparing outcomes between NC and AC in MIBC across major international centers involved in the treatment of urogenital tumors and with experience in this field. Nonetheless, it has several limitations that derive mainly from the retrospective nature of the study, a potential bias in the distribution of patient characteristics and the type of statistical analysis, which does not allow definitive conclusions. A major limitation in this comparison is that NC is administered based on clinical staging whereas AC is given based on pathologic staging, making the comparison even more difficult. There is always the potential for heterogeneity in outcomes and understaging based on clinical staging.

Another problem is the lack of data concerning performance status in all patients, and the heterogeneity of chemotherapy treatments, often without cisplatin (more than 20% of patients treated without cisplatin in each group). Moreover, subsequent therapies for metastatic disease, that may have affected OS and CSS results, were not available for all patients.

Ongoing trials such as the VESPER trial (NCT01812369) of perioperative chemotherapy for patients with locally advanced bladder cancer, have the aim of comparing the efficacy of GC and HD-MVAC in terms of PFS in patients treated with perioperative treatment, both before and after surgery. Another trial conducted by the SWOG will use the Co-eXpressionExtrapolatioN (COXEN) model to evaluate genetic profiling. Patients will be treated with either GC or HD M-VAC in the NCsetting (NCT02788201).

Moreover, the combination of multiple treatments, could be a way to achieve a better survival compared to surgery and perioperative chemotherapy. In a recent prospective randomized phase 2 study by Zaghloul et al. (19), AC with cisplatin and gemcitabine, was compared to the combination of AC plus radiotherapy, with a statistically significant improvement in locoregional recurrence free survival and a trend in better DFS and OS.

With new data arising from multiple TCGA analytical platforms, the hope in the future is that better molecular characterization of bladder cancers will help us to determine useful predictive factors to select patients who will benefit most from perioperative chemotherapy or other novel therapies (20).

## Author contributions

GB and CS designed the analysis and wrote the manuscript. DG conducted the statistical analysis and contributed to the writing of the manuscript. FC, EP, LH, EY, SC, SP, AA, TP, UD, NA, AB, SL, AN, UV, GN, JBe, JBa, and MG have made direct and intellectual contributions to the work and approved it for publication.

### Conflict of interest statement

EP: Advisory Board: AstraZeneca, Bristol-Myers Squibb, Clovis, Exelixis, Genentech/Roche, Horizon Pharma, Incyte, Inovio, Janssen; Research Funding: Merck, Novartis, Pfizer, Eli Lilly and Co., Acceleron. LH: Advisory: Bayer, Genentech, Dendreon, Pfizer, Medivation/Astellas, Kew Group, Theragene, Corvus, Merck, Exelixis; Research to the institution: Bayer, Sotio, Bristol-Myers Squib, Merck, Takeda, Dendreon/Valient, JannsenMedivation/Astellas, Genentech, Pfizer. EY: Consulting: Amgen, Bayer, Dendreon, Incyte, Janssen, Merck, QED, Seattle Genetics, Tolmar; Research funding to institution: Dendreon, Merck, Seattle Genetics. SC: Consulting/advisory: Roche, Clovis Oncology, Merck Research support: Astex Pharmaceuticals, Clovis Oncology. SP: Consulting: Genentech, Aveo, Eisai, Roche, Pfizer, Novartis, Exelixis, Ipsen, BMS, Astellas. TP: Honararia: Roche BMS AZ Merck; Research funding: AZ and Roche. UD: Advisory/Consultant: Bayer, Pfizer, Astellas, Janssen, Sanofi, BMS, Ipsen, Novartis. NA: Consultancy to Pfizer, Novartis, Merck, Genentech, Eisai, Exelixis, Clovis, EMD Serono, BMS, Astra Zeneca, Foundation One, Astellas, Ely Lilly, Bayer, Argos, Medivation, Clovis, Nektar; Research funding to my institution on my behalf: Active Biotech, Astra Zeneca, Bavarian Nordic, BMS, Calithera, Celldex, Eisai, Exelixis, Genetech, GSK (glaxosmithkline), Immunomedics, Janssen, Medivation, Merck, New link Genetics, Novartis, Pfizer, Prometheus, Rexahn, Sanofi, Takeda, Tracon. UV: Consulting: Bayer, Exelixis, BMS Inc.; Research support: Merck and Exelixis Inc.; GN: Research support4SC lectures: Pfizer, Pierre Fabre, Roche, MSD; advisory role: Roche, IMS Health, BMS, medac, MSD, Pfizer; travel grants: Roche Parma, Pfizer Pharma, BMS. JBe: Advisory: Bayer, Genentech, Pfizer, Medivation/Astellas, Merck, Asta-Zeneca, Exelixis; Research to the institution: Merck, Takeda, Pfizer. MG: Advisory Board: Merck, Genentech, BMS, Astra Zeneca, Pfizer; Research Funding: Novartis, Merck, Genentech, BMS, Astra Zeneca; CS: Research funding: Janssen, Lilly, Roche, Pfizer; Advisory Board: Lilly, BMS, Merck, Clovis, Pfizer. The remaining authors declare that the research was conducted in the absence of any commercial or financial relationships that could be construed as a potential conflict of interest.
