# Supplementary material for: Neoadjuvant vs. Adjuvant Chemotherapy in Muscle Invasive Bladder Cancer (MIBC): Analysis From the RISC Database
Source: Front Oncol. 2018 Nov 19;8:463. doi: 10.3389/fonc.2018.00463 (PMC6252384; doi:10.3389/fonc.2018.00463)

# APPENDIX

### **Supplementary Table 1**: Univariate and Multivariate Analysis of Patient Characteristics

#### DFS

|  | Univariate | Multivariate |
| --- | --- | --- |
| GENDER  (male vs  female) | 1.06 (0.82-1.37) P=0.68 |  |
| AGE  (>= 65 years vs < 65 years) | 1.11 (0.90-1.36) P=0.32 |  |
| CHARLSON  1-2 vs 0  >= 3 vs 0 | 0.95 (0.74-1.22) P=0.68  1.16 (0.88-1.54) P=0.29 |  |
| SMOKING HISTORY  Current vs never  Former vs never | 0.91 (0.68-1.22) P=0.53  0.86 (0.67-1.11) P=0.26 |  |
| pT  1-2 vs 0  3-4 vs 0 | 2.85 (1.75-4.66) P<0.0001  4.51 (2.89-7.05) P<0.0001 | 3.03 (1.82-5.05) P<0.0001  4.88 (3.01-7.89) P<0.0001 |
| pN  + vs 0 | 1.86 (1.49-2.33) P<0.0001 | 1.56 (1.21-2.01) P=0.001 |
| REGIMEN  Neoadjuvant vs adjuvant | 0.78 (0.63-0.96) P=0.02 | 1.75 (1.36-2.26) P<0.0001 |

#### CSS

|  | Univariate | Multivariate |
| --- | --- | --- |
| GENDER  (male vs  female) | 0.90 (0.63-1.27) P=0.54 |  |
| AGE  (>= 65 years vs < 65 years) | 0.88 (0.65-1.20) P=0.42 |  |
| CHARLSON  1-2 vs 0  >= 3 vs 0 | 0.84 (0.58-1.21) P=0.35  1.35 (0.92-1.98) P=0.12 |  |
| SMOKING HISTORY  Current vs never  Former vs never | 1.17 (0.77-1.77) P=0.45  0.88 (0.61-1.28) P=0.51 |  |
| pT  1-2 vs 0  3-4 vs 0 | 3.49 (1.62-7.53) P=0.001  5.11 (2.49-10.45) P<0.0001 | 4.82 (2.21-10.57) P<0.0001  7.99 (3.80-16.78) P<0.0001 |
| pN  + vs 0 | 1.46 (1.07-1.99) P=0.02 |  |
| REGIMEN  Neoadjuvant vs adjuvant | 1.06 (0.79-1.43) P=0.70 | 1.96 (1.41-2.73) P<0.0001 |

#### OS

|  | Univariate | Multivariate |
| --- | --- | --- |
| GENDER  (male vs  female) | 0.89 (0.66-1.20) P=0.45 |  |
| AGE  (>= 65 years vs < 65 years) | 1.01 (0.78-1.31) P=0.92 |  |
| CHARLSON  1-2 vs 0  >= 3 vs 0 | 1.02 (0.75-1.39) P=0.89  1.49 (1.07-2.07) P=0.02 |  |
| SMOKING HISTORY  Current vs never  Former vs never | 1.09 (0.77-1.56) P=0.62  0.84 (0.61-1.16) P=0.30 |  |
| pT  1-2 vs 0  3-4 vs 0 | 2.62 (1.44-4.77) P=0.002  3.75 (2.17-6.48) P<0.0001 | 3.43(1.86-6.30) P<0.0001  5.48 (3.09-9.71) P<0.0001 |
| pN  + vs 0 | 1.43 (1.09-1.87) P=0.009 |  |
| REGIMEN  Neoadjuvant vs adjuvant | 1.08 (0.83-1.39) P=0.57 | 1.79 (1.35-2.37) P<0.0001 |

### Supplementary Table 2: Neoadjuvant regimens in MIBC.


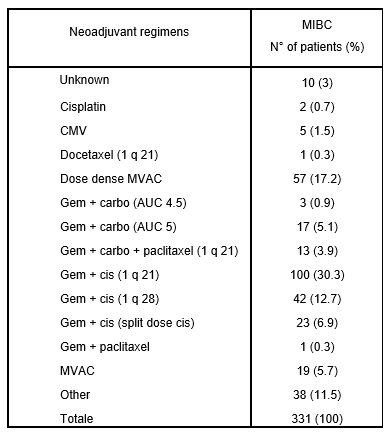


Abbreviations: CMV (Cisplatin, methotrexate, vinblastine); AUC (Area under the curve);

M-VAC (methotrexate, cisplatin, vinblastine, doxorubicin).

### Supplementary Table 3: Adjuvant regimens in MIBC.


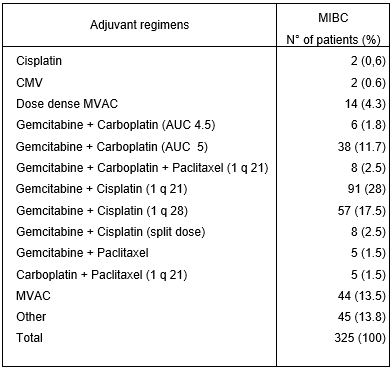


Abbreviations: CMV (Cisplatin, methotrexate, vinblastine); AUC (Area under the curve); M-VAC (methotrexate, cisplatin, vinblastine, doxorubicin)

### Supplementary survival curves: OS and DFS at 5 years between patients with complete pathologic response (pT0) after NC compared to those with residual disease (p T1-4).


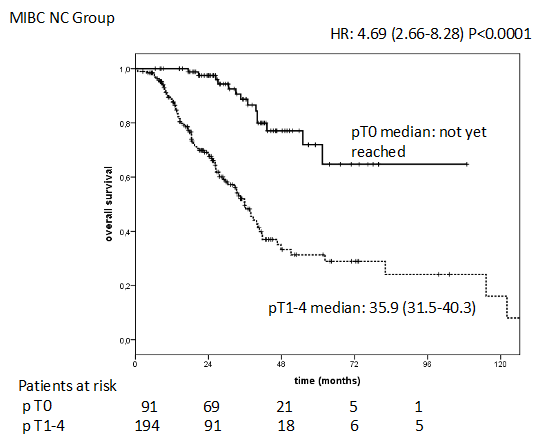


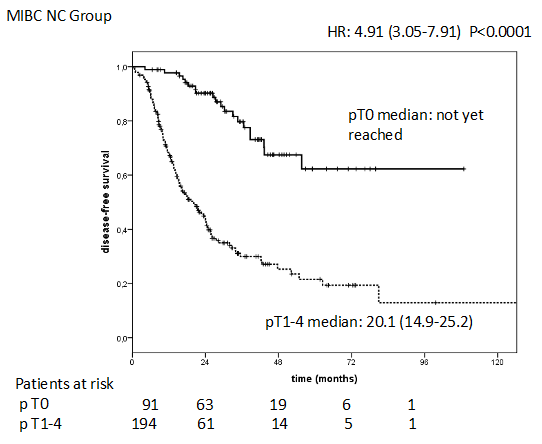


### Supplementary survival curves: DFS and OS at 5 years for NC in MIBC for patients without lymph node metastasis pN0, compared to those with lymph node involvement p N+.


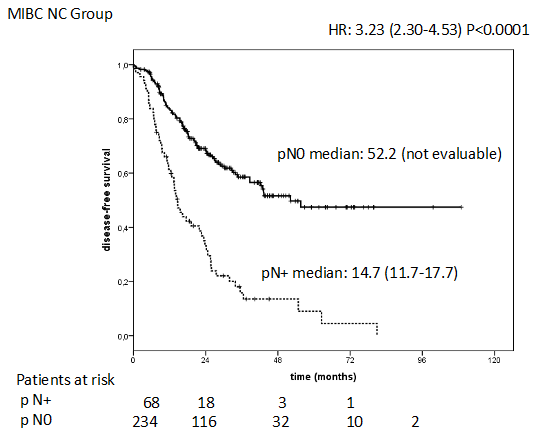


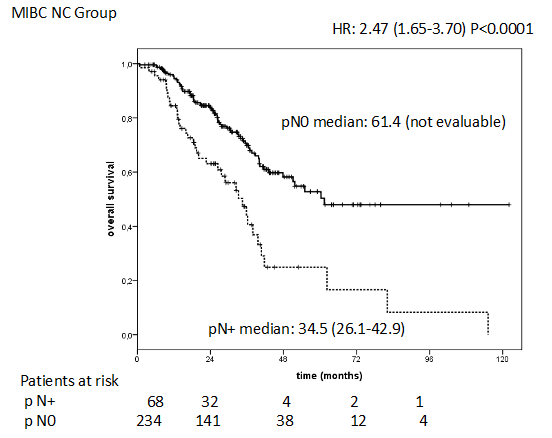

Supplement: Supplementary file 1 [file Data_Sheet_1.docx]
